# Modos de atuar a síndrome congênita do Zika vírus entre famílias da Bahia, Brasil

**DOI:** 10.1590/0102-311XPT235123

**Published:** 2025-04-11

**Authors:** Monica Machado de Matos, Litza Andrade Cunha, Jorge Alberto Bernstein Iriart, Luís Augusto Vasconcelos da Silva

**Affiliations:** 1 Universidade Federal da Bahia, Salvador, Brasil.

**Keywords:** Zika Vírus, Família, Ciências Sociais, Zika Virus, Family, Social Sciencies, Virus Zika, Familia, Ciencias Sociales

## Abstract

Esta investigação tem como objetivo apresentar uma rede de eventos na qual foi sendo tecida a síndrome congênita do Zika vírus (SCZ). Acompanhou-se de perto as necessidades de cuidado das famílias, os medos, as incertezas biomédicas, as dificuldades de entendimento, os desafios delineados nos momentos de se fazer escolhas difíceis e todo o fluxo de acontecimentos em que a realidade da doença foi sendo produzida nas diversas práticas. A orientação metodológica tomou como referência o pressuposto de que a SCZ emergiu à medida que foi sendo manipulada nas diferentes práticas em espaços diversos. Por conceber os processos de saúde e doença desse modo, este estudo se inspirou no trabalho da etnógrafa Annemarie Mol. Trata-se, portanto, de uma praxiografia, entendida como um método de pesquisa, que não se volta para a investigação de significados acerca da doença, mas como ela é atuada nas práticas. A doença trazida à condição de aparecimento não foi resultante apenas da atividade médica, mas as práticas de cuidado das famílias, suas experiências, percepções, descobertas e compreensões acerca da doença participaram da produção do que conhecemos hoje como SCZ. Nesta investigação buscou-se evidenciar como a síndrome foi trazida à condição de aparecimento por uma variedade de agentes, fazendo com que distintos modos de ler o corpo e de lidar com a doença coexistissem incorporados às várias atividades.

## Abstract (en)

This investigation aims to present a network of events in which congenital Zika syndrome (CZS) was woven. The study closely followed the care needs of the families, the fears, the biomedical uncertainties, the difficulties in understanding, the challenges outlined at the moment in which difficult decisions were made and the entire flow of events that show the reality of the disease. The methodological orientation was based on the assumption that CZS emerged as it was manipulated in different practices in various spaces as a reference. By conceiving the processes of health and disease in this way, this study was inspired by the work of ethnographer Annemarie Mol. It is, therefore, a praxiography, understood as a research method, which does not focus on the investigation of meanings regarding the disease, but on the practices in which it is acted out. The rise of the disease was not only the result of medical activity, but the care practices of the families, their experiences, perceptions, discoveries and understandings about the disease also contributed to the production of what we know today as CZS. In this investigation, we sought to show how CZS appeared as a result of the actions performed by a variety of agents, causing different ways of reading the body and dealing with the disease to coexist incorporated into the various activities.

## Resumen

Este estudio tiene como objetivo presentar una red de eventos en la cual se entrelazó el síndrome congénito del Zika (SCZ). Se monitorearon de cerca las necesidades de atención de las familias, los temores, las incertidumbres biomédicas, las dificultades de comprensión, los desafíos descritos en los momentos de toma de decisiones difíciles y todo el flujo de eventos en los cuales se estaba produciendo la realidad de la enfermedad en las diversas prácticas. La orientación metodológica se basó en el supuesto de que el SCZ surgió a medida que la manipulaba en distintas prácticas en diferentes espacios. Al considerar los procesos de salud y enfermedad de esta manera, este estudio se inspiró en el trabajo de la etnógrafa Annemarie Mol. Se trata de una praxiografía entendida como un método de investigación, que no recurre a una investigación de significados sobre la enfermedad, sino a las prácticas en las que actúa. La enfermedad en la condición de surgimiento no solo fue el resultado de la actividad médica, sino que las prácticas de atención de las familias, de sus experiencias, percepciones, descubrimientos y entendimientos sobre la enfermedad participaron en la conformación de lo que hoy conocemos como SCZ. Este estudio buscó mostrar cómo el SCZ fue llevado a la condición de surgimiento por una variedad de agentes al coexistir diferentes formas de evaluar el cuerpo y tratar la enfermedad incorporadas a diversas actividades.

## Introdução

No final do ano de 2015, famílias brasileiras se depararam com um acontecimento inédito: o aumento do nascimento de crianças apresentando perímetro cefálico não correspondente ao padrão conhecido e considerado normal. Em momentos inspiradores de cuidados como a gravidez, parto e puerpério, famílias transitaram por diversos espaços e conversaram com profissionais, pesquisadores e repórteres em busca de respostas esclarecedoras dos acontecimentos ^1^.

Enquanto as necessidades de cuidado, de compreensão e de fazer escolhas difíceis se delineavam no fluxo dos acontecimentos, a trajetória da síndrome congênita do Zika vírus (SCZ) foi emergindo, tecida na vida cotidiana de corpos e comunidades, tramada por distintos atores, produzida em uma diversidade de práticas.

O que se seguiu foi um encontro entre famílias, profissionais e pesquisadores, compartilhando sentimentos e testemunhando transformações. Mesmo diante de uma condição comum (a marca da atuação de um vírus), cada realidade era vivida de forma distinta. Diferentes tessituras resultaram do encontro com diversos modos de cuidar, expectativas, emoções e mudanças nas condições das crianças. Nesses mais de oito anos, modos específicos de lidar com esse acontecimento emergiram. Corpos foram lidos, leis foram instituídas, associações criadas, práticas de cuidado atualizadas e a vida de famílias transformada. Nesse fluxo, novas realidades foram moldadas, num encontro de muitas versões desse fenômeno.

Nesse turbilhão de experiências desestabilizadoras, persistia o incômodo provocado pela dificuldade de nomear ou reduzir os acontecimentos vividos por cada família a uma única nosologia. Foi nas tramas desse incômodo que esta pesquisa se realizou. O interesse deste estudo se dirigiu à investigação dos diversos elementos que participaram da produção da SCZ.

Em lugar de tomar como dada essa categoria biomédica, o presente trabalho buscou acompanhar os modos como a SCZ foi emergindo e passando a existir à medida que foi sendo manipulada nas diversas práticas, não apenas biomédicas, mas familiares. É por conceber os processos de saúde e doença desse modo que este estudo se inspirou nos trabalhos da etnógrafa Annemarie Mol ^2^ ^,^ ^3^ ^,^ ^4^. De acordo com os estudos, as doenças não têm uma realidade única e não antecedem as práticas sociais de onde emergem. Nesse sentido, a SCZ é resultante de entrelaçamentos de elementos e relações costuradas em dimensões vividas que se fazem existir por meio das práticas de diversos agentes ^2^ ^,^ ^3^ ^,^ ^4^ ^,^ ^5^ ^,^ ^6^. A proposta aqui apresentada se caracteriza por um mergulho nas práticas onde foram produzidos os modos de atuação do que se promulgou chamar de síndrome congênita do Zika vírus.

## Considerações teóricas e metodológicas

Do ponto de vista teórico e metodológico, a investigação se orientou pelos pressupostos da Teoria Ator-Rede, fundamentando-se principalmente nos trabalhos de Mol ^2^ ^,^ ^3^ ^,^ ^4^, Mol & Law ^5^ e Mol et al. ^6^. O ponto de partida foram as práticas de cuidado de famílias que participaram de um projeto de intervenção chamado *Juntos*, realizado entre junho de 2017 e junho de 2018 no Brasil. Voltado especialmente para famílias de crianças diagnosticadas com a SCZ vivendo em contextos com recursos econômicos limitados, o programa teve como objetivos ampliar conhecimentos e habilidades dos cuidadores para que pudessem lidar melhor com as crianças e incentivar a criação de redes de suporte na comunidade ^7^.

Os grupos eram conduzidos por uma dupla ^8^: sendo uma fisioterapeuta, uma fonoaudióloga e duas mães de crianças diagnosticadas com SCZ. O programa utilizava uma metodologia participativa baseada em comunidades e consistia em dez encontros semanais, nos quais famílias compartilhavam experiências e construíam juntas conhecimento prático ao longo de três meses. Os temas se relacionavam ao cuidado das crianças (posições e movimentos, comer e beber, comunicação, brincadeiras, atividades da vida diária etc.); relações com a comunidade, direitos, estigmas e inclusão; além de um foco específico em apoio emocional e no compartilhamento de informações sobre a SCZ.

Participaram do *Juntos* 35 famílias de crianças com até dois anos de idade, diagnosticadas pelos serviços de saúde (unidades de saúde e centros de reabilitação), em três cidades da Bahia, Brasil. Utilizou-se a categoria SCZ para descrever qualquer criança com sinais diretamente atribuídas ao vírus Zika.

A partir desse contato surgiu o interesse da presente pesquisa em aproximar-se da realidade de mulheres, homens e crianças nascidas com as marcas de um problema de saúde e diferentemente afetadas por ele. Por meio desse trabalho de grupos de apoio às famílias, foi possível acompanhar a construção coletiva de pessoas que ao estarem juntas, trazendo experiências individuais sobre dimensões do cuidado em suas práticas diárias, contribuíram para produção dessa realidade. Em alguns momentos foi preciso manejar situações difíceis, como, por exemplo, a morte de uma das crianças, além de testemunhar muitos relatos sobre questões profundas e desafiadoras que permeiam a vida.

Ao final dos grupos, o contato com as famílias e com os pesquisadores do *Juntos* foram mantidos e seguiram produzindo materiais, facilitando treinamentos, assim como mentorias sobre o programa. Foi possível também conhecer a realidade de famílias do Rio de Janeiro e Recife (Pernambuco), no Brasil, e Cali na Colômbia.

Vale ressaltar que o projeto foi um momento importante de imersão no contato com as crianças e permitiu acompanhar um fluxo de acontecimentos nos quais múltiplas realidades da SCZ foram feitas. Entretanto, o *Juntos* é um programa que pretendia apoiar as famílias nas práticas de cuidado, e teve, portanto, objetivos, processos de trabalho e resultados distintos da investigação aqui exposta. Os frutos do estudo apresentados aqui são resultantes da tentativa de engajamento em um modo de fazer pesquisa que buscou se debruçar sobre uma prática de maneira implicada e que se teceu enlaçada no encontro de muitos atores e de diversas fontes de informação como artigos, documentos, diário de campo, conversas, visitas, passeios, vídeos, observações, além dos dados produzidos a partir do *Juntos*. O foco principal se voltou para as práticas constitutivas dos modos de atuar a SCZ. Para escrever este trabalho, elegemos quatro famílias com as quais o contato foi mais próximo (Quadro 1). Essas e outras famílias participaram da produção do que chamamos de síndrome congênita do Zika.

**Quadro 1:** Caracterização das famílias.

FAMÍLIA	IDADE (ANOS)	PROFISSÃO	PRIMEIRO CONTATO COM A DOENÇA
Luna, Jéssica, Pedro	4, 23 e 30	Mãe: dona de casa Pai: trabalhador informal	No parto, a partir de sinais no corpo da criança. A doença ainda não tinha nome
Juca, Élen e Léo	3, 23 e 29	Mãe: estudante e vendedora informal Pai: operador de máquinas	Notícias na televisão, após nascimento do filho
Heitor, Júlia, Binho e Breno	2, 21, 25 e 5	Mãe: estudante Pai: vendedor informal	Notícias no jornal durante a gestação
Ravi, Larissa, Adriano e Diego	2, 30, 33 e 7	Mãe: merendeira Pai: trabalhador informal	Exames pré-natais

Fonte: elaboração própria.

“*A Teoria Ator-Rede é uma família díspare, de ferramentas, sensibilidades e métodos de análise materiais-semióticas que tratam tudo nos mundos social e natural como um efeito continuamente gerado das teias das relações dentro das quais estão localizadas. Nada tem realidade ou forma fora do engajamento nessas relações. Seus estudos exploram e descrevem as redes, as teias e práticas dessas relações*” ^9^ (p. 2).

Segundo Mol ^3^ e Mol & Law ^5^, não haveria uma doença autônoma, com existência própria e independente das práticas. A “doença” se faz atuada na prática a partir de agenciamentos, de interações, portanto, não se deve tomar o atalho e reduzir a doença aquilo que está sob a pele, escondida em um corpo. Dessa forma não há uma cisão entre uma doença concebida como uma entidade biológica, estado físico e as percepções sobre a doença, situadas nos contextos sociais e culturais. Essa cisão multiplicaria as perspectivas sobre a doença, sendo vista por distintos olhares e manteria o objeto (doença) intocado, como um campo de atuação exclusivo da medicina. Em lugar disso, os autores supõem a existência de uma rede de associações entre várias atuações da doença, que passa a ser produzida por atores heterogêneos em diversos espaços, a partir de uma interação complexa entre fisicalidades (biologia) e “o social” ^3^ ^,^ ^5^. 

Seguindo essas inspirações metodológicas adotamos aqui a praxiografia como um método de pesquisa, ressaltando que tal orientação não se volta para a investigação de significados acerca da doença, mas para as práticas em que ela atua. Ao se debruçar sobre as práticas nas quais as doenças são feitas, miramos as conexões locais entre as realidades e objetos, analisando os modos pelos quais vão sendo produzidas e ordenadas em arranjos múltiplos e heterogêneos que emergem em uma rede que não está pronta, mas que é feita e refeita nas práticas, cotidianamente por humanos e não humanos ^9^ ^,^ ^10^. 

Assim como um corpo vivo, que no intuito de manter-se unido e integrado está engajado num processo contínuo de se fazer nas práticas do dia a dia, a realidade de uma doença não está dada de antemão, mas envolve manipulações ^5^. Para Mol ^3^, não existe uma única ontologia, inerente à natureza das coisas e sim ontologias múltiplas trazidas à existência, sustentadas ou abandonadas por meio de práticas sociais cotidianas. Por essa razão a realidade é considerada múltipla. 

## Múltiplas realidades

Momentos de encontro com versões da SCZ se tornam marcadores significativos e são normalmente descritos como momentos “*devastadores*”, “*o dia em que sonhos e desejos desmoronam*”, “*que incertezas esmagam o coração*”. O contato das famílias com as múltiplas realidades da SCZ ocorreu em tempos, espaços e modos distintos que serão descritos a seguir.

Jéssica havia sido acompanhada durante toda a gestação e tudo estava dentro do esperado. Na hora do parto “*ouviu*” um silêncio total até que os médicos começassem a conversar entre si. Foi quando ainda em meio as emoções do parto, sentiu a “*vida virar de cabeça pra baixo*” ao ouvir que caso sua filha sobrevivesse “*seria incapaz de tudo*”. Saiu da sala de parto, sem muitas explicações, sentindo-se completamente atordoada, com o coração apertado. Jéssica se perguntava “*o que será de minha filha, o que será de mim?*”.

Após 15 dias, receberam alta. Em casa, uma grande surpresa. Apesar de chorar intensamente, logo nos primeiros meses, “*ela rolou na cama, se sentou e ficou em pé sozinha antes do tempo esperado*”. Até o sétimo mês não perceberam nenhuma alteração na filha, até que Luna começou a “*perder o que ela já tinha... ela perdeu o sentar, o rolar e o ficar de p*é”. 

Antes de ser decretada como um alerta global, famílias vivenciaram em suas casas a prática e o sentido da ciência doméstica ^11^, quando passaram a suspeitar que o choro desesperador que chamava a atenção de vizinhos não era apenas uma irritabilidade comum. Que talvez os filhos não reagissem aos chamados porque não escutavam ou enxergavam bem. Foi no corpo a corpo, que para muitas famílias a SCZ foi se revelando, ao perceber que seus filhos não se desenvolviam do modo esperado. A partir de sinais físicos na criança, novos modos de ler o corpo e de cuidar emergiram.

Élen estava no segundo ano da faculdade quando engravidou. É a filha mais velha de quatro irmãos. Seu irmão caçula tinha apenas um ano quando Juca nasceu. Durante o quarto mês sentiu coceiras no corpo. Chegou a ir para emergência, mas “*ninguém sabia o que era*”. Quando Juca nasceu, “*super saudável*”, foram realizados todos os exames no hospital e depois de dois dias tiveram alta. Em uma das várias noites sem dormir, viu nos noticiários a respeito de crianças que nasciam com perímetro cefálico inferior ao esperado. Lembra que naquele momento pensou nas famílias, mas não fez nenhuma associação com seu filho pois tudo estava bem. Por ter ajudado a cuidar dos irmãos mais novos, percebia diferenças no desenvolvimento de Juca, entretanto a realidade vivenciada em casa não lhe provocava preocupações. Amamentava e já estava introduzindo a alimentação sólida quando Juca aos cinco meses realizou uma sorologia que confirmou a presença do vírus Zika no seu sangue. Ela foi informada que o filho evoluía com atraso no desenvolvimento neuropsicomotor. Inicialmente, a notícia do atraso não lhe chegou como um peso. Até então, ela e seu filho levavam uma vida tranquila. O momento aterrorizante aconteceu um mês depois da confirmação da sorologia, impulsionado pelo conhecimento de uma série de informações que eram mais assustadoras do que a realidade vivida. O contato com o diagnóstico moldou um novo modo de ser.

“*No momento que eu ouvi essa palavra paralisia cerebral, foi aí que entrei em choque, porque eu já tinha visto fotos e conhecia crianças e aquilo era uma realidade diferente da que eu imaginava para meu filho. A partir desse diagnóstico me assustei. Então, ele não vai andar nem com três anos, nem falar com quatro, pode passar uma vida inteira sem andar ou falar* (...) *Eu tive que viver um luto*” (Élen).

O aumento do nascimento de crianças que apresentavam desproporção craniofacial em cidades do Nordeste brasileiro foi o alerta que chamou a atenção para o fato de que algo maior acontecia. Apesar de a microcefalia ter sido a marca mais evidente, a atuação dos exames na produção de versões da doença fez com que a SCZ atingisse seu apogeu quando os profissionais detectaram os danos cerebrais e as manchas de calcificação intracranianas, visíveis nos exames de imagem das mulheres grávidas. Em versão consonante com a tradição biomédica, a doença foi sendo produzida, ordenada e distribuída em práticas diversas, em distintas situações nos espaços de atuação da biomedicina (hospitais, laboratórios, maternidades e clínicas), a partir de diferentes técnicas ^1^ ^,^ ^12^. 

**Figura 1:**
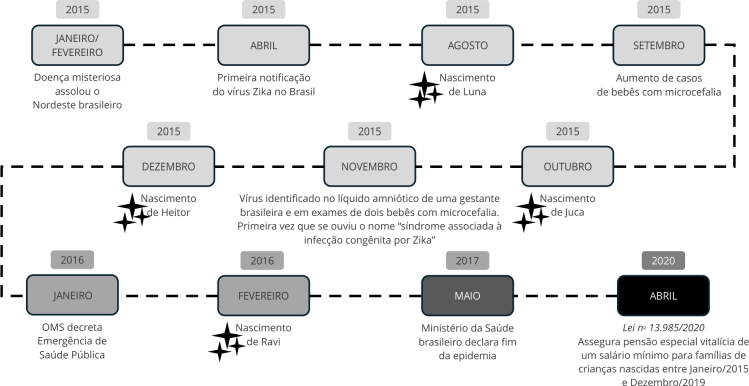
Linha do tempo.

Desde o momento em que foi confirmada a correlação entre a infecção por Zika vírus na gravidez e o nascimento de crianças que apresentavam microcefalia e outras alterações, as famílias contam que viveram dias difíceis. Boatos, fotos em jornais, exames e afirmações de pequena chance de sobrevivência das crianças contribuíram para ampliar a vivência de medo de famílias que gestavam, tentavam engravidar ou estavam cuidando de seus filhos. Impactadas com o que viam e escutavam, não sabiam o que fazer, nem o que poderia lhes acontecer. A SCZ estava sendo produzida. Entrelaçada as vivências familiares, suas versões científicas, políticas, sociais e midiáticas foram emergindo (Figura 1). 

OMS: organização Mundial da Saúde. Fonte: elaboração própria, com base em Diniz ^1^, Teixeira et al. ^12^, Ministério da Saúde ^18^ e Brasil ^19^.

“*Nos jornais só passava que as crianças estavam nascendo com má-formação congênita, não explicava direito o que era, só dizia que deveriam ter um cuidado maior. Eu comecei a evitar a assistir, aquilo me fazia muito mal porque eu já sabia que tinha tido Zika e todo mundo que sabia e via qualquer coisa que passava no jornal me chamava pra ver. Eu só chorava e me fechei. Eu não queria saber, porque pra mim estava tudo bem comigo e com meu filho, aquilo me fazia muito mal*” (Júlia).

“*Quando fui fazer o exame tava cheio de jornal, pois um dia antes, tinham descoberto a relação da Zika e a micro. Nos jornais não se falava outra coisa. Que podia ter autismo, que podia ter isso e aquilo, que morria, que já tinha muitos óbitos, eu não aguentava mais escutar, e eu estava ali fazendo exame esperando pra saber*” (Larissa).

Larissa foi facilitadora do *Juntos* e mora em uma cidade no interior da Bahia. Após receber o resultado do exame, ela e seu companheiro Adriano se sentiram desnorteados. Em busca de compreender o que acontecia, buscaram outra clínica para realizar nova ultrassonografia. Perguntou ao médico como o filho estava, se tinha pés e braços. O médico lhe informou que sim, mas que foram identificados danos cerebrais e que Ravi tinha as características das crianças com a má-formação. Ao questionar como seria quando o filho nascesse, o médico lhe mostrou com o celular uma foto “*e me disse que normalmente iria pra UTI, ficaria intubado, eu receberia alta e ele não*”.

No dia do nascimento de Ravi vivenciaram uma experiência singular: “*Quando ele nasceu, a médica colocou de costas pra mim, eu só via a cabeça, sem cabeça, né? E me disse: ‘você sabe, né, que ele é doentinho?’* (...) *Eu estava tendo meu segundo filho! A sensação era a mesma de quando tive Diego. Depois que a gente viu o rosto, ele não permitiu mais que a gente se perguntasse nada*” (Larissa).

O diagnóstico, as imagens aterrorizantes, os exames e os noticiários pareciam não corresponder ao que era experimentado por Larissa e Adriano diante da chegada de Ravi. Em versões da SCZ que se tentavam coordenar, foi na vivência das práticas tecidas dia após dia que as famílias foram contrapondo o que muitas vezes tinham escutado dos médicos sobre o futuro das crianças. A passagem do tempo foi fundamental para que compreendessem a complexa rede em que versões da SCZ iam sendo reveladas e como a partir das suas próprias atuações novas realidades poderiam emergir. Até o nascimento das crianças, o conhecimento existente sobre o Zika era insuficiente para que se pudesse prever sua extensão e desdobramentos. O aprimoramento da sensibilidade dos cuidadores em reconhecer as singularidades de cada criança e a ampliação da sua capacidade de ler os gestos e movimentos dos filhos possibilitaram que as famílias ganhassem confiança, constatando que a busca por essa atenção sintonizada ^6^ é fundamental para produção do cuidado e desenvolvimento dos filhos. Nesse sentido, a biomedicina também se alimentou das práticas e dos conhecimentos produzidos pelas famílias.

“*No início foi bem difícil, porque muitos médicos me amedrontaram dizendo que tinham crianças que não iam sobreviver nem seis meses de vida. Hoje o desenvolvimento de Heitor é surpreendente e dou graças a Deus pelo desenvolvimento dele e por ele ter chegado a minha família, pois através de Heitor nós crescemos muito como pessoas*” (Júlia).

“*Quando Ravi nasceu era tudo completamente novo, eu tinha filho de 8 anos de idade que era perfeito, então pra gente era tudo o médico* (...) *Hoje não, a gente que consegue lidar com ele. A gente sabe e percebe tudo. As caras dele eu já sei. Eu confio muito no que eu e Adriano sabemos. Porque Ravi nos ensinou que o médico não é o mestre e nem a última palavra. Acho muito importante o que eles me dizem, acolho e recebo, debato, coloco minha opinião, mas hoje temos segurança em dizer eu quero dessa forma, ou eu acho que não é necessário levar para o médico. Adquirimos essa confiança com o tempo... antes o que era tudo na mão do médico passou a ficar na nossa mão, não deixando o lado profissional, jamais, mas a gente que convive e vê todos os dias, isso fez a gente ter essa confiança que a gente tá certo e pode tomar a decisão*” (Larissa).

## Refazendo novas realidades

“*No começo é mais difícil, porque a gente não sabe como lidar. A gente não sabia o que era a microcefalia. Algumas sabiam que ia nascer especial. E depois que nasce, quando vai crescendo, algumas coisas vão piorando, outras vão melhorando. A maioria das crianças tem dificuldades pra se alimentar, a minha mesmo, no começo era bem difícil mas agora já superamos bastante. Ela tinha dificuldade pra beber água, pra se alimentar com pedaços, porque ela entalava, tossia, vomitava. Mas aos poucos fomos aprendendo*” (Jéssica).

Desde o nascimento, as crianças necessitam de cuidados específicos. Trata-se de corpos que se movem, se comunicam, se alimentam, aprendem e se engajam em atividades do dia a dia de modo distinto. Nesse sentido, a temporalidade do cuidado e a longitudinalidade da atenção que marcam as situações de cronicidade fizeram com que a vida das famílias se transformasse de modo a se ajustar às necessidades das crianças. Isso significou uma série de adaptações; a inclusão de um conjunto de demandas, como idas frequentes às consultas e realização de exames; busca por equipamentos de reabilitação e a adaptação desses equipamentos; luta por direitos; participação em grupos; manejo de medicações, dietas e monitoramento de sintomas; e realização de atividades de estimulação, enfim, práticas diárias de cuidado com as crianças, consigo, com a casa e com a família ^13^ ^,^ ^14^ ^,^ ^15^.

Em um processo contínuo de cuidado, como numa dança onde passos avançam e retornam, as famílias foram produzindo suas próprias ciências. Ao desenvolverem diversas estratégias para cuidar dos filhos, foram experimentando encontrar seus próprios ritmos, estabelecendo relações, incorporando o exterior e “excorporando” ações ^4^. Aos poucos passaram a reconhecer os tipos de choro, descobrir as melhores posições para alimentar, dar banho, conversar, as brincadeiras mais engraçadas. Ao estimularem os filhos com o melhor de suas possibilidades, em suas próprias formas, apesar da imprevisibilidade do corpo, as famílias inventaram alternativas que fizessem sentido e fossem adequadas às suas realidades. Ao vivenciarem as práticas do cotidiano, famílias atuavam a SCZ, criando modos para se viver melhor.

Para Larissa e Adriano, um momento difícil foi quando Ravi tinha menos de um ano de idade e o médico lhe prescreveu uma cadeira de rodas. Inicialmente, rechaçaram a ideia, “*era como se tirasse a possibilidade do sim e deixasse o não*”. Mesmo percebendo que Ravi apresentava alguns atrasos no desenvolvimento, as incertezas apareciam como uma oportunidade de se concentrar na qualidade do presente e sonhar com o futuro, pois na prática também desfrutavam de momentos de bem-estar. 

“*Não sabíamos direito como ia ser. Ele era mais molinho, chorava absurdamente, tinha irritabilidade e crises convulsivas. Tudo era diferente, não segurava o pescoço, mas, até um ano de idade, não achava a limitação tão grande, a medicação acalmou as crises*... *Até os sete meses ele mamava tudo, foi tranquilo, a adaptação para mamadeira, colher. As coisas de fato só se tornaram mais complexas, após um ano. Até você compreender é bem doido e difícil*” (Larissa).

Entretanto, com o passar dos meses que se seguiram, ao vivenciarem as práticas do cotidiano, notaram que ao carregar Ravi no colo, ele demonstrava desconforto.

“*Ele já estava mais pesado, estávamos vendo que não fazia bem. Ele já estava ficando tortinho pra um lado, que era o jeito que a gente pegava. Começamos a sentir a necessidade de uma cadeira para que ele ficasse bem posicionado, tivesse mais autonomia*” (Larissa).

Apesar de a aceitação da cadeira de rodas para Larissa e Adriano não ter sido fácil, gradualmente, foram ajustando as emoções em relação à chegada desse dispositivo nas suas vidas, avaliando os benefícios, conversando entre si e com outras famílias que viviam conflitos semelhantes. Vendo na prática como aquela cadeira poderia ampliar as possibilidades do filho.

Winance ^16^ chama de processo de ajustamento esse trabalho compartilhado que emerge de um corpo estendido que é simultaneamente objeto e sujeito de cuidado. A cadeira de rodas atua e a partir dela emerge um corpo coletivo. Os familiares, a cadeira de rodas, além de outras “vozes” humanas e não humanas que “falam”, atuam em uma rede de relações, em que diferentes versões do corpo e da doença existem articuladas às práticas que estão envolvidas ^4^. Os elos que uniram Ravi, os pais e a cadeira estão sempre em transformação, criando e inventando novas alternativas de ação em um processo permanentemente inacabado, onde as novas possibilidades que se abrem convivem com a emergência de impossibilidades ^6^ ^,^ ^16^.

Ao descermos as escadas da casa e caminharmos nas ruas com Ravi, sentimos como o tempo para se deslocar é diferente. A ação de se mover por onde existe desorganização urbana, dificuldades com os transportes públicos e adaptações insuficientes, leva as famílias a se habituarem à ideia de que para se deslocar precisam não apenas pensar no melhor trajeto, mas contar com seres humanos e não humanos. Sentem na pele o quanto a marca da SCZ transforma seu modo de viver e os coloca frente a inúmeras barreiras na sociedade, revelando a SCZ que não está em um corpo físico, mas em uma série de materialidades que a produzem.

“*Hoje eu penso no lugar que eu vou. Eu vou conseguir chegar lá sozinha com meu filho? Como é* [o] *chão do lugar que eu vou? Nesse prédio tem muito degrau ou é mais fácil de entrar?* (...) *aí a doença me aparece e eu me sinto incapaz, quando vejo as dificuldades* (...) *eu queria fazer tudo que ele precisa e não precisar ser fora da minha cidade. Tentamos cuidar dele como uma criança que não tem nada, mas essas coisas me fazem me sentir incapaz*” (Larissa).

Chama a atenção como é comum na vida das famílias lidar com versões da SCZ que chegam a partir de olhares e reações. “*A gente tem que se acostumar*”. Larissa e outros familiares contam como se sentem entristecidos e lamentam ao perceber que pessoas parecem sentir pena delas e das crianças por focarem apenas nas diferenças, tomando suas vidas como histórias únicas de tragédia ^17^, imaginando que por conta da SCZ levam uma vida apenas de dificuldades. Percebem que algumas pessoas são exageradamente solícitas e gentis, outras ignoram, fingem que não escutam ou que não veem, recuam, parecem sentir medo, “*como se fosse algo contagioso*”. Contam como é comum estarem com amigos em um momento descontraído e às vezes o amigo chorar. “*Às vezes, o que as pessoas acham que é um fardo muito grande, para gente é tranquilo, a gente faz com todo carinho e com todo amor. É o contrário*” (Larissa).

## Tecendo redes

Quando o corpo não funciona exatamente da maneira como se espera, conduzir a dança que caracteriza a relação entre os filhos e seus pais desafia ainda mais as expectativas e práticas daqueles que cuidam, produzindo tensões que envolvem a dependência de cuidados, a qualidade das redes sociais, a importância e a necessidade de referências e apoios para construção de autonomia e do que se faz necessário para que os filhos se tornem sujeitos no mundo.

O presente difícil e o futuro incerto ^13^ fazem com que as histórias não sejam tramas lineares e muitas vezes objetivos e desejos particulares são substituídos por uma abertura para possibilidades ainda desconhecidas. Lidar com incertezas sobre quais decisões tomar quando as escolhas envolvem riscos ou questionamentos acerca do que na prática é um bom cuidado faz parte da vida das famílias. Dúvidas não são evitadas, nem impedem a ação, ao contrário, são tecidas, discutidas por diversas pessoas, em muitos espaços, envolvendo práticas situadas e materialidades heterogêneas ^4^.

Foi a partir do nascimento dos filhos que muitas famílias se conectaram, dando início a jornadas que apesar de singulares, são partilhadas. No intuito de superar os desafios impostos e melhorar as condições de vida, as famílias buscam tirar o máximo de proveito das experiências de seus pares ^14^. Nesse sentido, todas elas relatam a importância de participarem de grupos que emergiram a partir dos vínculos estabelecidos tanto com os serviços e profissionais de saúde, como também de maneira informal. 

Apesar das exclusões vivenciadas, onde muitas vezes as próprias relações familiares se tornam mais restritas, devido a abandonos e distanciamentos, a partir da interação entre pessoas que conhecem os problemas uns dos outros de forma “real”, se conforma uma zona de pertencimento e confiança. Além da diminuição da sensação de isolamento e obtenção de ajuda prática, a partir dos elos estabelecidos as famílias ampliam seu núcleo familiar vivenciando como parte de si as dores e as conquistas dos outros.

“*A gente se tornou uma família. Temos vínculo desde que nossos filhos são pequenos, um fortalece o outro. Todas as vezes que a gente sabe que uma criança com microcefalia morre, pra gente é uma dor imensa… a gente sente o baque*” (Jéssica).

“*Eu vi que não é somente eu que estou nessa situação. Criamos um grupo de apoio entre nós, onde cada uma compartilha as dificuldades, os ganhos, os nossos sentimentos, quando bons ou ruins. A gente criou uma família. Às vezes, para minha família eu tinha que me manter forte! E no grupo eu vi que não é só eu que passo por isso. E aí eu me senti, assim, mais forte ainda! Foi ótimo falar da gente em si, fora ser mãe de Juca... como nós também!*” (Élen).

As redes sociais virtuais se firmaram como estratégia para o enfrentamento dos desafios cotidianos. Das mais diversas regiões, foi por meio dos computadores e celulares que o vivido em cada universo particular se transmitiu a outros, entrelaçando histórias e produzindo novas redes. Através das trocas com os pares, famílias constroem soluções coletivas, atuam em processos de luta por seus direitos. Seja em grupos virtuais ou nas vivências cotidianas, as famílias se acolhem, refletem sobre suas práticas, ressignificam suas experiências e se nutrem de esperança.

“*Eu ficava com medo, pelo preconceito, ficava com medo de deixar ela na escola, se quem pegasse ela pra tomar conta rejeitasse ou machucasse* (...) *As meninas foram me incentivando, dizendo que a filha estava evoluindo, eu fui pensar mais. Hoje eu já levo ela, boto ela sentada na escolinha e ela fica. Eu hoje estou me sentindo segura, sei que minha filha é capaz daquilo*” (Jéssica).

Ao poder escutar e falar sobre seus desafios, as famílias relatam que se sentem menos culpadas e mais confiantes. É na vivência das práticas em conjunto que as pessoas vêm tecendo laços que as tem possibilitado vivenciar as aventuras do desenvolvimento humano e as contingências da vida, desfrutando de um convívio em que ter uma boa vida e sentir prazer são possíveis. Embora considerem suas jornadas desafiadoras, famílias passam a questionar o que é a SCZ, enfatizando que apesar da imprevisibilidade do corpo, do presente difícil e do futuro incerto, se sentem felizes.

“*As crianças nos inspiram a ser humildes, a fazer uma autoanálise da nossa existência. O que de fato tem valor pra gente. O que de fato é importante. Eu acho que ela veio pra confundir os egoístas e trazer humanidade pra gente*” (Adriano).

## Considerações finais

Em uma tentativa de ultrapassar o tradicional limite das fronteiras disciplinares em que se pressupõe a anterioridade ontológica das doenças, esta investigação buscou explorar os modos de atuação da SCZ que estiveram envolvidos nas diversas maneiras em que ela foi sendo produzida. Utilizando o conceito de realidades múltiplas, a presente pesquisa se debruçou sobre práticas de famílias, nas quais as distintas SCZ emergiram em uma rede que não estava pronta, mas que foi sendo feita no cotidiano. Diante das suas múltiplas performances, o estudo seguiu uma trilha, mirando possíveis interconexões entre suas versões, ordenadas em arranjos heterogêneos e encenadas na vida, onde vários atores participaram da trama. O foco foi observar seus próprios modos de atuar que resultaram em realidades múltiplas.

Mesmo na tradição biomédica, onde o substrato da SCZ foi decretado no corpo físico, este estudo demonstrou que as várias versões nem sempre se somaram formando um todo coerente, foram muitas as maneiras pelas quais ela foi colocada em prática. Corpos foram medidos, observados, diversos materiais foram manipulados e variaram de um local para o outro. A SCZ foi vista em sangues, fluidos corporais, exames, e o nome “síndrome congênita do Zika” foi usado para diferentes objetos: volume cerebral diminuído, calcificações intracranianas, atraso no desenvolvimento, entre outros.

Entretanto, antes de tornar-se uma preocupação mundial e ganhar versões na mídia, nas práticas de tratamento e na política, esse fenômeno havia tocado cidades, lares. Famílias vivenciaram um acontecimento importante e transformador de suas vidas, doaram seus corpos para compreender o que acontecia e ao atuarem suas práticas nos apresentaram múltiplas realidades. Hoje seguem performando modos de viver com ela. As práticas de cuidado são processos abertos, se fazem e refazem a partir de regulações, acordos, apostas envolvendo muitas coisas e pessoas. Foi no corpo a corpo, lidando com um recém-nascido no fluxo da vida que as práticas de cuidado das famílias, suas percepções, descobertas e compreensões participaram da produção do que hoje é a SCZ. 

A partir de práticas de famílias esta pesquisa buscou versões da SCZ que não estão localizadas apenas no corpo físico ou em locais de saúde, mas que se fizeram mutuamente, evidenciando que a SCZ foi emergindo não apenas a partir das importantes descobertas da biomedicina, mas também por uma variedade de pessoas e uma multiplicidade de ações, fazendo com que distintos modos de ler o corpo e de lidar com as situações possam coexistir incorporados às várias atividades. 

Faz-se importante destacar que, ao conceber a multiplicidade da SCZ, esta investigação não buscou seus distintos significados ou interpretações, negligenciando a sua biologia, nem priorizou os seus aspectos psicossociais, separando-os de sua fisicalidade. Pois, para aqueles que se depararam com o inesperado e difícil acontecimento e foram lançados em processos de transformação de suas vidas, lares e sonhos, a microcefalia não está separada da dificuldade para se comunicar, as atrofias não estão separadas da ida das crianças à escola, nem a disfagia está separada da colher, do ativismo ou das reinvenções, mas emergem juntas. 

Ao olhar hoje para uma criança que vai para escola ou brinca em um parque, contrariando as previsões iniciais, se vê como ações de sujeitos fazem surgir novas realidades. É, portanto, um convite para que outros modos de concepção do real sejam explorados, onde as diferentes práticas nem sempre resultam em elementos que se encaixam perfeitamente expressando uma síntese definitiva ou singular, como muitas vezes se supõe.
